# Track-to-Track Association for Intelligent Vehicles by Preserving Local Track Geometry

**DOI:** 10.3390/s20051412

**Published:** 2020-03-04

**Authors:** Ke Zou, Hao Zhu, Allan De Freitas, Yongfu Li, Hamid Esmaeili Najafabadi

**Affiliations:** 1 Key Laboratory of Intelligent Air-Ground Cooperative Control for Universities in Chongqing, College of Automation, Chongqing University of Posts and Telecommunications, Chongqing 400065, China; zhuhao@cqupt.edu.cn (H.Z.); liyongfu@cqupt.edu.cn (Y.L.); 2 Department of Electrical, Electronic and Computer Engineering, University of Pretoria, Hatfield 0002, South Africa; allan.defreitas@up.ac.za; 3 Department of Electrical and Computer Engineering, University of Calgary, Calgary, AB T2N 1N4, Canada; Hamid.esmaeili@gmail.com

**Keywords:** Gaussian mixture model, local track geometry, maximum likelihood estimation, sensor bias, track association

## Abstract

Track-to-track association (T2TA) is a challenging task in situational awareness in intelligent vehicles and surveillance systems. In this paper, the problem of track-to-track association with sensor bias (T2TASB) is considered. Traditional T2TASB algorithms only consider a statistical distance cost between local tracks from different sensors, without exploiting the geometric relationship between one track and its neighboring ones from each sensor. However, the relative geometry among neighboring local tracks is usually stable, at least for a while, and thus helpful in improving the T2TASB. In this paper, we propose a probabilistic method, called the local track geometry preservation (LTGP) algorithm, which takes advantage of the geometry of tracks. Assuming that the local tracks of one sensor are represented by Gaussian mixture model (GMM) centroids, the corresponding local tracks of the other sensor are fitted to those of the first sensor. In this regard, a geometrical descriptor connectivity matrix is constructed to exploit the relative geometry of these tracks. The track association problem is formulated as a maximum likelihood estimation problem with a local track geometry constraint, and an expectation–maximization (EM) algorithm is developed to find the solution. Simulation results demonstrate that the proposed methods offer better performance than the state-of-the-art methods.

## 1. Introduction

Reliable situational awareness plays an essential role in intelligent vehicles and surveillance systems [1,2,3,4,5,6]. Typical intelligent vehicles employ various types of sensors, such as radio detection and ranging (radar), light detection and ranging (lidar), and video. The radar sensor determines the relative location and the radial velocity of objects by emitting radio signals. Radar measurements often consist of false alarm detections in addition to detections from real objects or targets while missing some target-originated detections. The lidar sensor uses laser light to detect objects. Compared to radar, it provides more detailed measurements at an increased cost. Video sensors are feature-rich with a wide field-of-view, but they are more sensitive to different illumination and weather conditions [1]. Since these sensors have different sensing capabilities, features, and accuracies, the use of multiple heterogeneous sensors can result in more reliable and multi-modal environment perception systems. Therefore, pedestrians, vehicles, and obstacles are typically detected and tracked using a multi-sensor system in intelligent vehicles [7,8,9]. A multi-sensor multi-target tracking module jointly estimates the states and the number of targets from sensor measurements in intelligent vehicles, and it can be broadly categorized as centralized or distributed. The advantage of the distributed tracking systems is that they can provide a degree of scalability and robustness not achievable by traditional centralized tracking systems [1].

**Figure 1:**
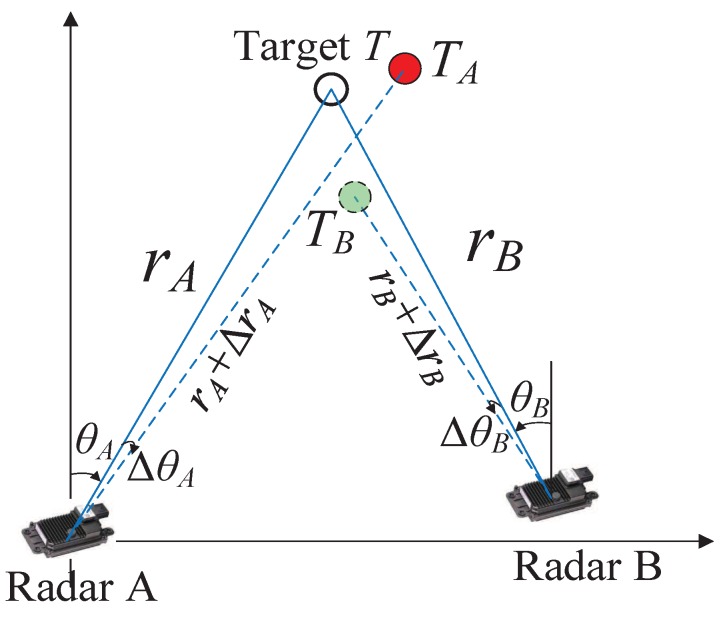
Relationship between sensor bias and local tracks.

Track-to-track association (T2TA) is a crucial task in distributed tracking to find the correspondence between local tracks from different sensors. It is commonly applied to combine the local tracks of a sensor with those of another sensor to form the global tracklist. For automotive applications, radar, lidar, and video sensors in environmental perception systems for intelligent vehicles use different coordinate systems and sampling frequencies. Therefore, a spatio-temporal calibration should be performed to align the detections from different sensors [1]. In practice, detection from radar, lidar, and video sensors cannot always be calibrated or aligned accurately [10]. Each sensor may cover a different part of the surveillance region with a detection probability of less than one. As a result, some local tracks from a sensor may not correspond to those of other sensors. The range, azimuth, and elevation biases of a radar sensor may lead to errors in the local tracks from that sensor. The relationship between radar sensor bias and local tracks is presented in Figure 1, where two radar sensors, *A* and *B*, and one target, *T*, are shown. The radar sensor bias leads to the reporting of the target *T* as tracks $T_{A}$ and $T_{B}$ by radar *A* and *B*, respectively. Note that if the biases in *A* and *B* radars are significant, the distance between $T_{A}$ and $T_{B}$ is correspondingly high. In this case, $T_{A}$ and $T_{B}$ is considered as originating from two different targets by the T2TA module. Therefore, T2TA in intelligent vehicles or surveillance systems suffers from many challenges, including missing detection and measurement bias. In this paper, the focus is on the problem of independent T2TA for each frame in the presence of missed detections and sensor bias.

To formulate the T2TA as an optimization problem, different statistical distances or metrics are proposed in literature [11,12,13,14,15,16,17,18,19,20,21,22,23,24]. In [11], a weighted statistical distance is proposed for T2TA with the assumption that the local estimation error of one sensor is independent of those of other sensors for the same target. In [13], the independence assumption is relaxed, and a modified statistical distance with dependent errors is developed for T2TA. In [19], three algorithms based on the squared Mahalanobis distance are investigated, and the nearest neighbor (NN) and global nearest neighbor (GNN) algorithms are applied to compute the distance between two tracks for T2TA. In [20], a likelihood function for T2TA from multiple sensors is derived, and the multidimensional assignment algorithm is employed to solve the optimal matching problem. State augmentation data, which combines the kinematic state information and the additional feature state information, is proposed to perform T2TA in [21]. In [25], a track association algorithm is proposed based on the permutation matrix to support the track-to-track multi-sensor data fusion for multiple targets in an autonomous driving system. It is worth noting that most T2TA algorithms in intelligent vehicles employ the conventional GNN algorithm [25,26,27,28].

Nevertheless, most of the above methods do not consider the presence of sensor bias. In reality, T2TA performance significantly degrades with sensor bias [29]. Literature addressing this problem can be roughly divided into batch and online approaches. The batch approach is an offline implementation that estimates the track association and sensor bias using all local tracks [30,31,32]. A joint sensor registration and track-to-track fusion method is derived using an equivalent measurement method in [30], while a pseudo-measurement approach is adopted to handle registration and track fusion simultaneously in [31]. In [32], a joint registration, data association, and fusion method in a distributed sensor network is formulated as a maximum likelihood (ML) optimization problem. An expectation-maximization (EM) algorithm is then proposed to perform the ML optimization, joint association, and bias removal through following an iterative strategy. However, these methods are susceptible to being trapped in local minima and have high computational costs. The online approach is a real-time implementation to perform track association with sensor bias. In [33], relative position information among neighboring tracks is analyzed, and a reference topology feature is derived for the absolute position information. An optimal sub-pattern assignment (OSPA) metric is also proposed to construct the association cost for T2TASB. In [34], the OSPA metric is modified by compensating for the relative azimuth bias. In [35], the T2TASB is formulated as a point set registration problem, and a coherent point drift (CPD) algorithm is proposed to perform T2TASB. In the CPD algorithm, local tracks of one sensor are represented by Gaussian mixture model (GMM) centroids [4,36], where local tracks of all sensors are fitted to those of a reference sensor. Still, the CPD algorithm only exploits the relationship among local tracks from different sensors, i.e., it does not utilize the relative geometric relationship between a local track and its neighbors from each sensor. The geometry among neighboring local tracks is usually stable at least for a while and thus helpful in improving the T2TASB. Here, the geometry is inspired by the idea that the relationship between a local track and its neighbors from different sensors could be preserved after the transformation. Hence, the geometry among neighboring local tracks is usually stable at least for a while and thus helpful in improving the T2TASB.

In this paper, the problem of independent T2TA for each frame in the presence of missed detections and sensor bias is considered. A probabilistic method, called the local track geometry preservation (LTGP) algorithm, is proposed to handle T2TASB. In the proposed method, the local tracks of one sensor are represented by GMM centroids, and the local tracks of the other sensor are fitted to those of the first using a nonlinear transformation function. The local track geometry with *k*-connected neighborhood is developed, and the T2TASB is formulated as an ML optimization problem with an EM algorithm being proposed to address it.

Different from other literature, the main contributions of this paper are as follows:

1. The mathematical formulation for T2TASB is presented. Moreover, the local track geometry with *k*-connected neighborhood is derived to improve the robustness and accuracy of T2TASB. The proposed method extends the CPD method by considering the geometric relationship between neighboring tracks.
2. An EM algorithm is proposed for T2TASB. The optimal T2TASB correspondence matrix and transformation function between local tracks are estimated simultaneously.
3. The performance of the proposed method is validated by the experiments and computer simulations using the KITTI dataset.

This paper is organized as follows. The formulation of T2TASB is presented in Section 2. In Section 3, the EM algorithm is used to estimate the parameters in the proposed method. The performance of the proposed approach is evaluated using computer simulations and experiments on the KITTI dataset in Section 4 and Section 5, respectively. Finally, conclusions and future work are discussed in Section 6.

## 2. A New Method for T2TASB

In this section, the T2TASB problem is formulated and a new solution is proposed. Let $X_{k}^{s}$ denote the local tracks from sensor *s* at time *k*, where $X_{k}^{s}={\left[{{x_{1,k}^{s}}^{T},{x_{2,k}^{s}}^{T}\ldots ,x_{N_{k}^{s},k}^{s}}^{T}\right]}^{T}$. The  $x_{i,k}^{s}$ denotes the *i*-th track state estimate and the corresponding covariance from sensor *s* at time *k*, where k=1,2,…K, $i=1,2\ldots ,N_{k}^{s}$ with *K* and $N_{k}^{s}$ being the total number of discrete time steps and the number of tracks at time *k* by sensor *s*, respectively. Here, two sensors are applied to find the global track states, i.e., s=1,2. The objective of the T2TASB algorithm is to find the correspondence between $X_{k}^{1}$ and $X_{k}^{2}$.

In [35], T2TASB is considered as a probability density estimation problem. In this paper, the relative geometry among neighboring local tracks from each sensor is proposed to formulate a maximum likelihood estimation problem with a local track geometrical constraint. Assuming that the local tracks of one sensor are represented by GMM centroids, the corresponding local tracks of the other sensor are fitted to those of the first sensor. Let $x_{t,k}^{1}$ be the *t*-th data and $x_{l,k}^{2}$ be the centroid of the *l*-th component. That is,

(1) $$p\left(x_{t,k}^{1}\right)=\sum_{l=1}^{N_{k}^{2}}\pi_{t,l}^{k}N(x_{t,k}^{1}\left|f\left(x_{l,k}^{2}\right),\sigma_{k}^{2}I_{D}\right),$$

where N denotes the Gaussian distribution; $\sigma_{k}^{2}$ denotes the equal isotropic covariance at time *k*; *f* denotes the nonrigid transformation; I is the identity matrix; *D* is the size of a local track vector, and $\pi_{t,l}^{k}$ is the mixing coefficient at time *k* with $\sum_{l}\pi_{t,l}^{k}=1$. We introduce an indicator $Z^{k}=\left[z_{1}^{k},z_{2}^{k},\ldots z_{N_{k}^{1}}^{k}\right]$, where $z_{t}^{k}$ is a $1\times N_{k}^{1}$ binary vector with elements $z_{t,l}^{k}$ for $l=1,2,\ldots N_{k}^{2}$. The $z_{t,l}^{k}$ satisfy $z_{t,l}^{k}\in \left\{0,1\right\}$ and $\sum_{l}z_{t,l}^{k}=1$ conditions. That is, only one element in vector $z_{t}^{k}$ is 1 while all other elements are 0. We have

(2) $$p\left(z_{t}^{k}\left|\pi^{k}\right.\right)=\prod_{l=1}^{N_{k}^{2}}{\pi_{t,l}^{k}}^{z_{t,l}^{k}},$$

(3) $$p\left(x_{t,k}^{1}\left|x_{l,k}^{2},\sigma^{2},z_{t}^{k}\right.\right)=\prod_{l=1}^{N_{k}^{2}}N{\left(x_{t,k}^{1}\left|f\left(x_{l,k}^{2}\right),\sigma_{k}^{2}I_{D}\right.\right)}^{z_{t,l}^{k}},$$

where $\pi^{k}={\left\{\pi_{t,l}^{k}\right\}}_{t=1,2,\ldots N_{k}^{1}}^{l=1,2,\ldots N_{k}^{2}}$. Here, a distribution $\frac{1}{N_{k}^{1}}$ with weight *w* is employed to represents the component of a target detected by sensor 1, but not detected by sensor 2. The relationship between the local track lists $x_{t,k}^{1}$ and $x_{l,k}^{2}$ can be given by

(4) $$p\left(x_{t,k}^{1}\right)=w\frac{1}{N_{k}^{1}}+\left(1-w\right)\sum_{l=1}^{N_{k}^{2}}\pi_{t,l}^{k}N(x_{t,k}^{1}\left|f\left(x_{l,k}^{2}\right),\sigma_{k}^{2}I_{D}\right).$$

The nonrigid transformation *f* aligns the local tracks, while some nonlinear functions might be employed to approximate it as well. More detail of the nonrigid transformation is given in Appendix A. Here, the displacement function is adopted as [35]

(5) $$f\left(X_{k}^{2}\right)=X_{k}^{2}+G_{k}W_{k},$$

where $W_{k}$ is an $N_{k}^{2}\times D$ dimensional weight matrix of the Gaussian kernel; $G_{k}$ denotes an $N_{k}^{2}\times N_{k}^{2}$ Gaussian kernel matrix with elements $g_{ij}=e^{-\frac{1}{2\beta }{\left(x_{i,k}^{2}-x_{j,k}^{2}\right)}^{T}\left(x_{i,k}^{2}-x_{j,k}^{2}\right)}$; and, β denotes the width parameter in the smoothing Gaussian filter. To enforce the smoothness of transformation *f*, the constraint on the weight matrix $W_{k}$ can be given by [35,37]:

(6) $$E\left(W_{k}\right)=\mathrm{Tr}\left(W_{k}^{T}G_{k}W_{k}\right),$$

where Tr(.) denotes the trace of a matrix and superscript *T* denotes transposition. By ignoring the constants independent of $\left\{\sigma_{k}^{2}\right\}$ and $\left\{W_{k}\right\}$, the objective function of T2TASB can be written as

(7) $$\begin{array}{c} Q=\frac{D}{2}\sum_{t=1}^{N_{k}^{1}}\sum_{l=1}^{N_{k}^{2}}q(z_{t,l}^{k})\log\sigma_{k}^{2}+\frac{1}{2\sigma_{k}^{2}}\sum_{t=1}^{N_{k}^{1}}\sum_{l=1}^{N_{k}^{2}}q(z_{t,l}^{k})\times \;\;\left[{\left(x_{t,k}^{1}-f\left(x_{l,k}^{2}\right)\right)}^{T}\left(x_{t,k}^{1}-f\left(x_{l,k}^{2}\right)\right)\right]+\alpha \mathrm{Tr}\left({W_{k}}^{T}G_{k}W_{k}\right), \end{array}$$

where α controls the trade-off parameter and $q(z_{t,l}^{k})$ is used to denote $p(z_{t,l}^{k}=1\left|X_{k}^{1},X_{k}^{2}\right.)$.

Consider the membership probability $\pi_{t,l}^{k}$ in (2), which is assumed to be the same for all components [35]. Here, $\pi_{t,l}^{k}$ is initialized using the traditional nearest neighbor (NN) method [38] as follows:

(1) If track *t* from sensor 1 is associated with track *l* from sensor 2 at time *k* using NN assignment, we have

(8) $$\pi_{t,i}^{k}=\left\{\begin{array}{c} \tau \;\;\;\;\;\;\;\;\;\;\;\;\;\;\;\;\;\;\;\;\;\;\;\;\;\;\;\;\;\;\;\;\;\;\mathrm{if}\;\;\;i=l\\ \frac{1-\tau }{N_{k}^{2}-1}\;\;\;\;\;\;\;\;\;\;\;\mathrm{if}\;\;\;i\neq l \end{array}\right.,$$

where 0≤τ≤1 is the confidence in association with the NN method.

(2) If track *t* from sensor 1 is not associated with any track from sensor 2 at time *k* using NN assignment, then the uniform membership probability is applied:

(9) $$\pi_{t,l}^{k}=\frac{1}{N_{k}^{2}}\;\;l\in \left(1,2\ldots N_{k}^{2}\right).$$

**Figure 2:**
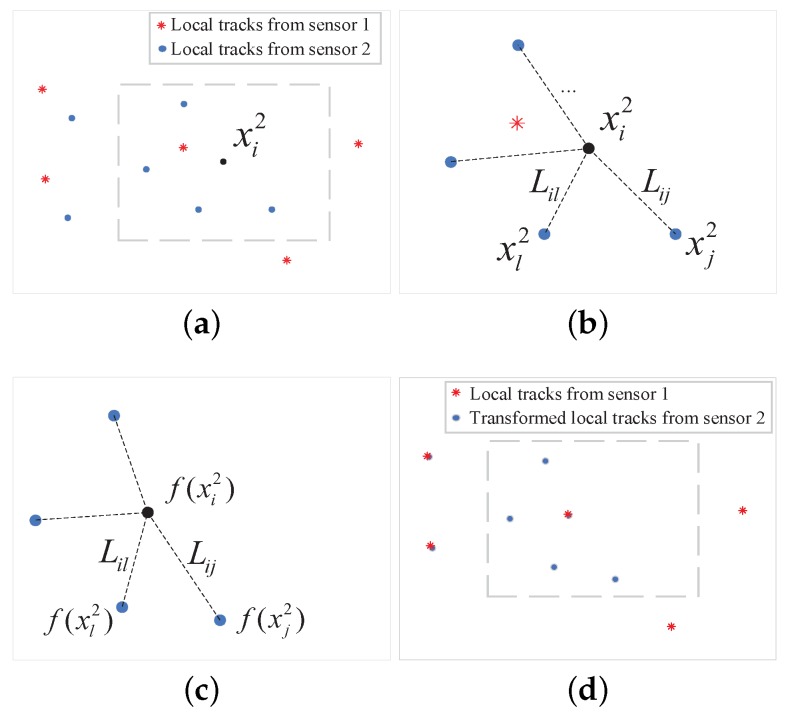
Schematic illustration of the geometrical constraint. (**a**) with local tracks from sensor 1 and 2, assign neighbors to each local track from its sensor, e.g., the four local tracks around ${x_{i}}^{2}$ (**b**) compute the weights L (**c**) perform the transformation *f* with the constraint that each local track ${x_{i}}^{2}$ be reconstructed by its neighbors with weights L after the transformation (**d**) align the Local tracks from sensor 1 and 2 after transformation *f* by maximizing the objective function.

The transformation *f* uses the relationship between local tracks from different sensors, but does not consider the relative geometry between one track and its neighbors from each sensor. The geometry is inspired by the idea that the relationship between a local track and its neighbors from different sensors could be preserved after the transformation, as depicted in Figure 2. To ensure an accurate T2TA, a geometrical constraint on the local tracks is proposed in this paper. A schematic illustration of the geometrical constraint is given in Figure 2.

We desire to preserve the geometry of tracks $X_{k}^{2}$ after the nonrigid transformation *f*. Based on the Euclidean distance between each local track and its neighbors in $X_{k}^{2}$, the *M* nearest neighbors of each local track in $X_{k}^{2}$ are obtained. Then, each point in $X_{k}^{2}$ is represented as a weighted linear combination of its *M* nearest neighbors. Let $L={\left\{L_{lj}\right\}}_{l=1:N_{k}^{2}}^{j=1:N_{k}^{2}}$ be an $N_{k}^{2}\times N_{k}^{2}$ weighted matrix. If track state $x_{j,k}^{2}$ does not belong to the *M* nearest neighbors of track state $x_{l,k}^{2}$, then $L_{lj}$ is set to 0. Here, matrix L is obtained by minimizing the following cost function:

(10) $$e\left(L\right)=\sum_{l=1}^{N_{k}^{2}}∥x_{l,k}^{2}-\sum_{j=1}^{N_{k}^{2}}L_{lj}x_{j,k}^{2}∥,$$

where the sum of each row of L is equal to 1. After the nonrigid transformation, the local track geometry can be preserved by minimizing the transformed cost function:

(11) $$\begin{array}{c} \begin{array}{cc} E(L) & =\sum_{l=1}^{N_{k}^{2}}q\left(z_{t,l}^{k}\right){∥f\left(x_{l,k}^{2}\right)-\sum_{j=1}^{N_{k}^{2}}L_{lj}f\left(x_{j,k}^{2}\right)∥}^{2}\\ \; & =\sum_{l=1}^{N_{k}^{2}}q\left(z_{t,l}^{k}\right){∥x_{l,k}^{2}+G_{k}\left(l,.\right)W-\sum_{j=1}^{N_{k}^{2}}L_{ij}\left(x_{j,k}^{2}+G_{k}\left(j,.\right)W\right)∥}^{2}, \end{array} \end{array}$$

where $G_{k}\left(i,.\right)$ is the *i*-th row of $G_{k}$. The objective function of T2TA with sensor bias in (7) is given by

(12) $$\begin{array}{c} \begin{array}{cc} Q_{1}\; & =Q+rE(L)\\ \; & =\frac{D}{2}\sum_{t=1}^{N_{k}^{1}}\sum_{l=1}^{N_{k}^{2}}q(z_{t,l}^{k})\log\sigma_{k}^{2}+\frac{1}{2\sigma_{k}^{2}}\sum_{t=1}^{N_{k}^{1}}\sum_{l=1}^{N_{k}^{2}}q(z_{t,l}^{k})\times \\ \; & \;\;\left[{\left(x_{t,k}^{1}-f\left(x_{l,k}^{2}\right)\right)}^{T}\left(x_{t,k}^{1}-f\left(x_{l,k}^{2}\right)\right)\right]+\alpha \mathrm{Tr}\left({W_{k}}^{T}G_{k}W_{k}\right)\\ \; & +\gamma \sum_{l=1}^{N_{k}^{2}}q\left(z_{t,l}^{k}\right){∥x_{l,k}^{2}+G_{k}\left(l,.\right)W-\sum_{j=1}^{N_{k}^{2}}L_{ij}\left(x_{j,k}^{2}+G_{k}\left(j,.\right)W\right)∥}^{2}, \end{array} \end{array}$$

where γ controls the trade-off between *Q* and E(L).

## 3. EM Solution for the Proposed Method

Let $\Theta =\left\{\left\{Z_{k}\},\{\sigma_{k}^{2}\},\{W_{k}\right\}\right\}$ be the unknown parameters. To obtain an ML estimate of Θ, the EM algorithm is applied here. There are two steps in the EM algorithm:

1). *E*-step: $E_{L}\left(\Theta ,\Theta^{\left(m\right)}\right)=Q_{1}$
2). *M*-step: $\Theta^{\left(m+1\right)}=\max E_{L}\left(\Theta ,\Theta^{\left(m\right)}\right)$,

where *m* is the iteration number of the algorithm. The *E*-step calculates the conditional expectation using the current estimate $\Theta^{\left(m\right)}$, whereas the *M*-step provides an updated estimation, $\Theta^{\left(m+1\right)}$. The estimate of Θ is updated by iterating through these two steps while the complete data likelihood function is maximized.

### 3.1. E-Step

First, $q(z_{t,l}^{k})$ can be found using Bayes’ theorem as

(13) $$q\left(z_{t,l}^{k}\right)=\frac{\pi_{t,l}^{k}N\left(x_{t,k}^{1}\left|f\left(x_{l,k}^{2}\right),\sigma_{k}^{2}I_{D}\right.\right)}{\underset{l}{\sum }\pi_{t,l}^{k}N\left(x_{t,k}^{1}\left|f\left(x_{l,k}^{2}\right),\sigma_{k}^{2}I_{D}\right.\right)+\frac{w}{1-w}\times \frac{N_{k}^{2}}{N_{k}^{1}}}.$$

### 3.2. M-Step

Then, $E_{L}\left(\Theta ,\Theta^{\left(m\right)}\right)$ is rewritten as

(14) $$\begin{array}{c} \begin{array}{cc} E_{L}\left(\Theta ,\Theta^{\left(m\right)}\right)\; & =\frac{1}{2\sigma_{k}^{2}}\left\{\mathrm{Tr}\left({\left(X_{k}^{1}\right)}^{T}diag\left(R_{k}1\right)X_{k}^{1}\right)\right.-2\mathrm{Tr}\left({\left({R_{k}}^{T}X_{k}^{1}\right)}^{T}\left(X_{k}^{2}+G_{k}W_{k}\right)\right)\\ \; & \left.+\mathrm{Tr}\left({\left(X_{k}^{2}+G_{k}W_{k}\right)}^{T}diag\left({R_{k}}^{T}1\right)\left(X_{k}^{2}+G_{k}W_{k}\right)\right)\right\}\\ \; & +\frac{D}{2}\log\left(\sigma_{k}^{2}\right)\sum_{t=1}^{N_{k}^{1}}\sum_{l=1}^{N_{k}^{2}}q(z_{t,l}^{k})+\alpha \mathrm{Tr}\left({W_{k}}^{T}G_{k}W_{k}\right)\\ \; & +\gamma \mathrm{Tr}\left({\left(X_{k}^{2}\right)}^{T}B_{k}X_{k}^{2}\right)+2\gamma \mathrm{Tr}\left({\left(X_{k}^{2}\right)}^{T}B_{k}G_{k}W_{k}\right)\\ \; & +\gamma \mathrm{Tr}\left({W_{k}}^{T}G_{k}B_{k}G_{k}W_{k}\right), \end{array} \end{array}$$

where diag(.) indicates diagonal matrix; $R_{k}$ is an $N_{k}^{1}\times N_{k}^{2}$ matrix with elements $q(z_{t,l}^{k})$ for $t=1,2,\cdots N_{k}^{1}$, $l=1,2,\cdots N_{k}^{2}+1$; $B_{k}={\left(I-L\right)}^{T}diag\left({R_{k}}^{T}1\right)\left(I-L\right)$; 1 represents the all-one column vector of corresponding length; and, I means the identity matrix.

The estimates of $\sigma_{k}^{2}$ and $W_{k}$ are iteratively updated by solving the corresponding partial derivative of the expected log likelihood to zero. That is,

(15) $$\begin{array}{c} \begin{array}{cc} \frac{\partial E_{L}\left(\Theta ,\Theta^{\left(m\right)}\right)}{\partial \sigma_{k}^{2}} & =-\frac{1}{2\sigma_{k}^{4}}\left\{\mathrm{Tr}\left({\left(X_{k}^{1}\right)}^{T}diag\left(R_{k}1\right)X_{k}^{1}\right)\right.\\ \; & -2\mathrm{Tr}\left({\left({R_{k}}^{T}X_{k}^{1}\right)}^{T}\left(X_{k}^{2}+G_{k}W_{k}\right)\right)\\ \; & \left.+\mathrm{Tr}\left({\left(X_{k}^{2}+G_{k}W_{k}\right)}^{T}diag\left({R_{k}}^{T}1\right)\left(X_{k}^{2}+G_{k}W_{k}\right)\right)\right\}\\ \; & +\frac{D\sum_{t=1}^{N_{k}^{1}}\sum_{l=1}^{N_{k}^{2}}q(z_{t,l}^{k})}{2\sigma_{k}^{2}}=0. \end{array} \end{array}$$

This results in

(16) $$\begin{array}{c} \begin{array}{cc} \sigma_{k}^{2} & =\frac{1}{D\sum_{t=1}^{N_{k}^{1}}\sum_{l=1}^{N_{k}^{2}}q(z_{t,l}^{k})}\left\{\mathrm{Tr}\left({\left(X_{k}^{1}\right)}^{T}diag\left(R_{k}1\right)X_{k}^{1}\right)\right.\\ \; & -2\mathrm{Tr}\left({\left({R_{k}}^{T}X_{k}^{1}\right)}^{T}\left(X_{k}^{2}+G_{k}W_{k}\right)\right)\\ \; & \left.+\mathrm{Tr}\left({\left(X_{k}^{2}+G_{k}W_{k}\right)}^{T}diag\left({R_{k}}^{T}1\right)\left(X_{k}^{2}+G_{k}W_{k}\right)\right)\right\}. \end{array} \end{array}$$

Similarly,

(17) $$\begin{array}{c} \begin{array}{cc} \frac{\partial E_{L}\left(\Theta ,\Theta^{\left(m\right)}\right)}{\partial W_{k}}\; & =\frac{1}{2\sigma_{k}^{2}}\left(-2G_{k}{R_{k}}^{T}X_{k}^{1}+2G_{k}diag\left({R_{k}}^{T}1\right)X_{k}^{2}\right.\\ \; & \left.+2G_{k}diag\left({R_{k}}^{T}1\right)G_{k}W_{k}\right)\\ \; & +2\alpha G_{k}W_{k}+2\gamma G_{k}B_{k}X_{k}^{2}+2\gamma G_{k}B_{k}G_{k}W_{k}=0. \end{array} \end{array}$$

Thus, $W_{k}$ can be obtained by solving

(18) $$\begin{array}{c} \left(diag\left({R_{k}}^{T}1\right)G_{k}+2\alpha \sigma_{k}^{2}I+2\sigma_{k}^{2}\gamma B_{k}G_{k}\right)W_{k}={R_{k}}^{T}X_{k}^{1}-diag\left({R_{k}}^{T}1\right)X_{k}^{2}-2\sigma_{k}^{2}\gamma B_{k}X_{k}^{2}. \end{array}$$

Here, $C^{k}$ is used to denote the cost matrix of T2TASB at time *k* as an $N_{k}^{1}\times \left(N_{k}^{2}+1\right)$ matrix with (t,l) element $C^{k}\left(t,l\right)$ for $t=1,2,\cdots N_{k}^{1}$, $l=1,2,\cdots N_{k}^{2}+1$ given by

$$C^{k}\left(t,l\right)=\left\{\begin{array}{c} -\mathrm{In}\left(\left(1-w\right)\pi_{t,l}^{k}N\left(x_{t,k}^{1}\left|f\left(x_{l,k}^{2}\right),\sigma_{k}^{2}I_{D}\right.\right)\right)\;\;\;\mathrm{if}\;\;l=1,2,\ldots ,N_{k}^{2}\\ -\mathrm{In}\left(w\frac{1}{N_{k}^{1}}\right)\;\;\;\;\;\;\;\;\;\;\;\;\;\;\;\;\;\;\;\;\;\;\;\;\;\;\;\;\;\;\;\;\;\;\;\;\;\;\;\;\;\;\;\;\;\;\;\;\;\;\;\;\;\;\;\;\;\;\;\;\;\;\;\;\;\;\;\;\;\;\;\;\;\;\;\;\;\;\mathrm{if}\;\;l=N_{k}^{2}+1 \end{array}\right.,$$

where $C^{k}\left(t,N_{k}^{2}+1\right)$ represents the cost of not making an assignment. The assignment of track *t* from sensor 1 to track *l* from sensor 2 can occur only if $C^{k}\left(t,l\right)<C^{k}\left(t,N_{k}^{2}+1\right)$ for $t=1,2,\cdots N_{k}^{1}$, $l=1,2,\cdots N_{k}^{2}$ with $C^{k}\left(t,N_{k}^{2}+1\right)$ being a gate. If that gate is violated, no assignment option is selected. The solution for the above assignment problem is computed using the Hungarian algorithm [39].

The proposed LTGP method for T2TASB is summarized in Algorithm 1.
**Algorithm 1** Proposed LTGP method for T2TASB**Require:**     Local tracks $X_{k}^{1}$ and $X_{k}^{2}$, parameters *w*, α, β, γ, *M*.1:Initialize $W_{k}=0$, $R_{k}=I_{N_{k}^{1}\times N_{k}^{2}}$.2:Search for the *M* nearest neighbors for each local track in $X_{k}^{2}$.3:Find L by minimizing (10).4:**while** not converged **do**5:*E*-step:6:   1. Assign membership probability $\pi_{t,l}^{k}$ using (8) or (9).7:   2. Update $q(z_{t,l}^{k})$ using (13).8:*M*-step:9:   1. Update $\sigma_{k}^{2}$ based on (16).10:   2. Update $W_{k}$ by solving the linear system in (18).11:**end while****Ensure:**     Transformed local track from sensor 2 is $f\left(X_{k}^{2}\right)=X_{k}^{2}+G_{k}W_{k}$.      Association matrix for T2TASB is $C^{k}\left(t,l\right)$ as in (3.2).

## 4. Computer Simulations

In this section, the performance of the proposed methods is evaluated using simulated data. Thirty targets following a discretized nearly constant velocity motion model [40] are tracked by multiple radar sensors. The initial target positions are randomly generated in the region $\left[-100\;\;\;\mathrm{km},100\;\;\;\mathrm{km}\right]\times \left[-100\;\;\;\mathrm{km},100\;\;\;\mathrm{km}\right]$. The initial velocities of these targets are chosen as [0.5km/s,0.2km/s]. The covariances of the process and measurement noise components are respectively set to $\mathrm{diag}\left(10^{-4}\;\;\;km^{2},10^{-4\;\;\;km^{2}}/s^{2},10^{-4}km^{2},10^{-4\;\;\;km^{2}}/s^{2}\right)$, and diag(10^−4^ km^2^, 10^−5^ rad^2^), where the cross-covariance terms have been ignored in the former [40]. The clutter is generated uniformly over the surveillance region using a Poisson random variable with a mean of 30 at each time step. The sampling period of the measurements is 1s. The number of time steps is 100.

**Figure 3:**
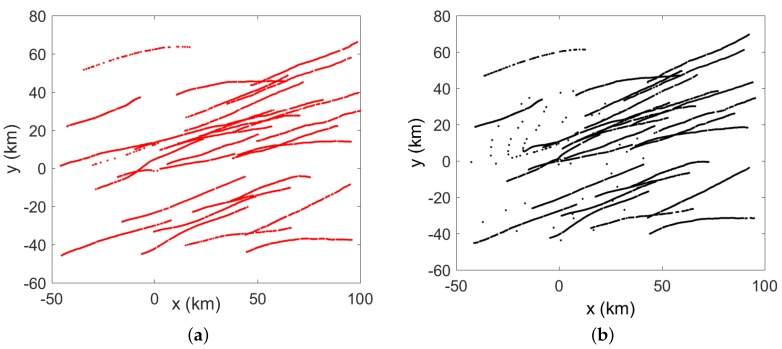
(**a**) Local tracks from sensor 1 (**b**) Local tracks from sensor 2.

Two radar sensors are considered in the distributed sensor network. The biases in the two sensors are set to $\eta_{1}={\left[1\;\;\;\mathrm{km},-0.017\;\;\;\mathrm{rad}\right]}^{T}$, and $\eta_{2}={\left[-2\;\;\;\mathrm{km},0.034\;\;\;\mathrm{rad}\right]}^{T}$. The detection probabilities $P_{d}$ of both radars are chosen as 0.95. Measurement-to-track association is performed at each sensor without considering the sensor bias. The local tracks from sensor 1 and sensor 2 are illustrated in Figure 3.

Parameter τ denotes the confidence in the association by the NN method. Parameter *w* denotes the initial assumption on the number of false targets detected by sensor 1, but not detected by sensor 2. Parameter β represents the width of the smoothing Gaussian filter in the nonlinear transformation function. Parameter *M* represents the number of nearest neighbors used in linear reconstruction to preserve the local track structure, while ρ is the parameter in the cross-covariance fusion. We set τ = 0.5, *w* = 0.2, β = 0.1, *M* = 10, and ρ = 0.4 throughout this paper.

**Figure 4:**
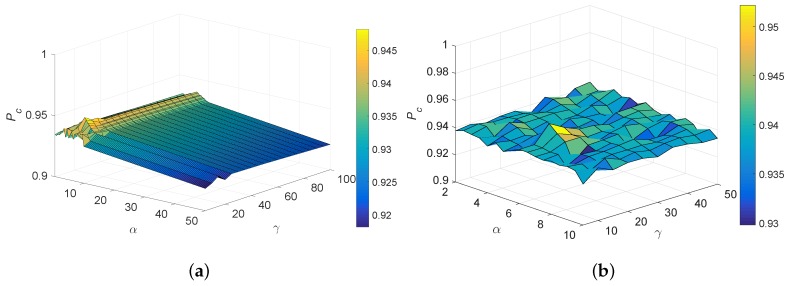
Model selection of the regularization parameters α and γ. (**a**) α∈[0.5,50] and γ∈[5,100], (**b**) α∈[2,10] and γ∈[5,50].

Parameters α and γ represent the trade-off regularization terms. The ranges of these parameters were determined experimentally. The correct association probability $P_{c}$ defined as the ratio of the correctly assigned tracks over the total number of tracks is employed as the primary metric for performance evaluation. The variation of $P_{c}$ with regularization parameters α and γ at time step *k* = 50 is shown in Figure 4. It is observed that the proposed method performs best when α∈[5,7] and γ∈[10,20]. Here, we set α = 6, and γ = 15.

**Figure 5:**
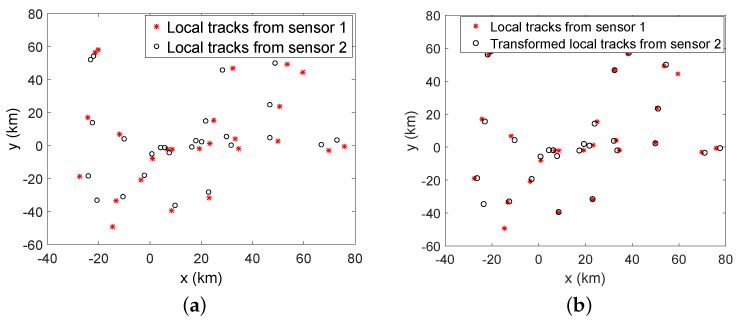
(**a**) Local tracks at time step *k* = 50 (**b**) Local tracks at time step *k* = 50 after transformation with proposed method.

The proposed LTGP algorithm is used for T2TASB, and the results at time step *k* = 50 are given in Figure 5, which illustrates that the local tracks from the two sensors are associated correctly by the proposed LTGP method.

**Figure 6:**
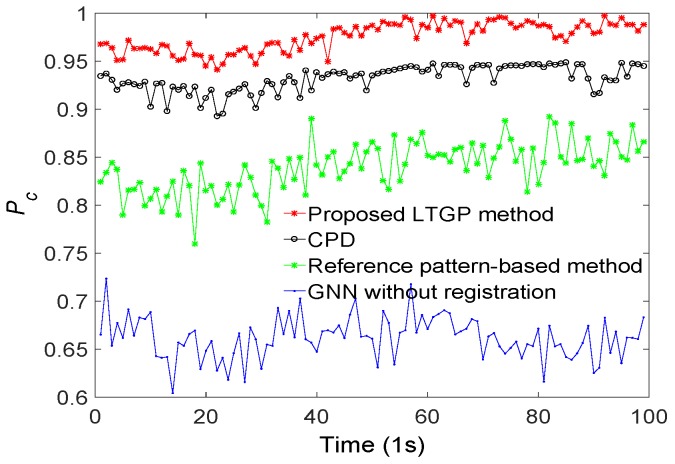
Correct association probabilities of GNN without registration, reference pattern-based algorithm, CPD algorithm and the proposed method.

**Figure 7:**
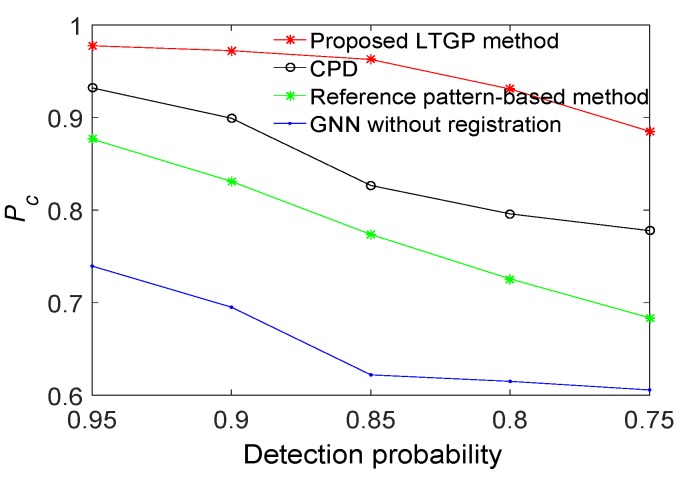
Correct association probabilities of GNN without registration, reference pattern-based algorithm, CPD algorithm and the proposed method for different detection probabilities.

**Figure 8:**
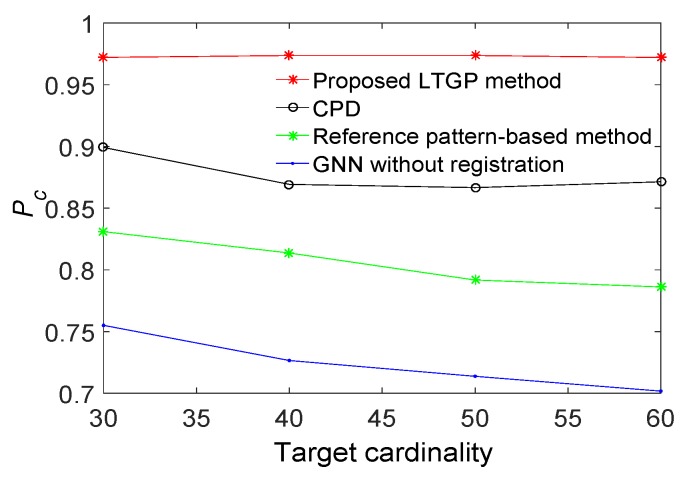
Correct association probabilities of GNN without registration, reference pattern-based algorithm, correlation-based algorithm and the proposed method for a detection probabilities of $P_{d}=0.9$ when target cardinality changes within a fixed surveillance region.

The performance of the proposed method is demonstrated next relative to those of GNN without registration, the reference pattern-based algorithm [33], and the CPD algorithm [35]. All results are averaged over 50 Monte Carlo runs. The proposed method achieves the best performance, as illustrated in Figure 6. Compared to the reference pattern-based algorithm, the CPD algorithm improves the $P_{c}$ by about 8%. The $P_{c}$ of the proposed method has improved by 5% as compared with the CPD algorithm. Furthermore, the results for a scenario with varying detection probabilities and different numbers of targets are respectively illustrated in Figure 7 and Figure 8. It is observed that the proposed algorithm outperforms the other three benchmark algorithms. From Figure 7, the performance of GNN without registration, reference pattern-based algorithm, and CPD algorithm degrade rapidly with a decreased detection probability. From Figure 8, the performance of the proposed method is almost constant while increasing the number of targets. Moreover, the average $P_{c}$ of the proposed method is improved by approximately 9% as compared with the CPD algorithm.

The computational complexities of the proposed LTGP algorithm are analyzed next. For simplicity, the same number of local tracks *N* for each sensor every time is considered. At each time step in the LTGP algorithm, the computational complexity to search the *M* nearest neighbors for each local track in $X_{k}^{2}$ is O((M+N)logN), using the *k*-d tree [41]; the computational complexity to obtain matrix L is $O(M^{3}N)$; and, the complexity of the EM algorithm is almost $O(N^{3})$ [42]. The computational complexity at each time step in the LTGP algorithm is $O(N^{3})$. Therefore, the total computational complexity of the proposed LTGP algorithm is $O(N^{3}K)$, where *K* is the total number of measurement samples.

## 5. Experiments on KITTI Dataset

**Figure 9:**
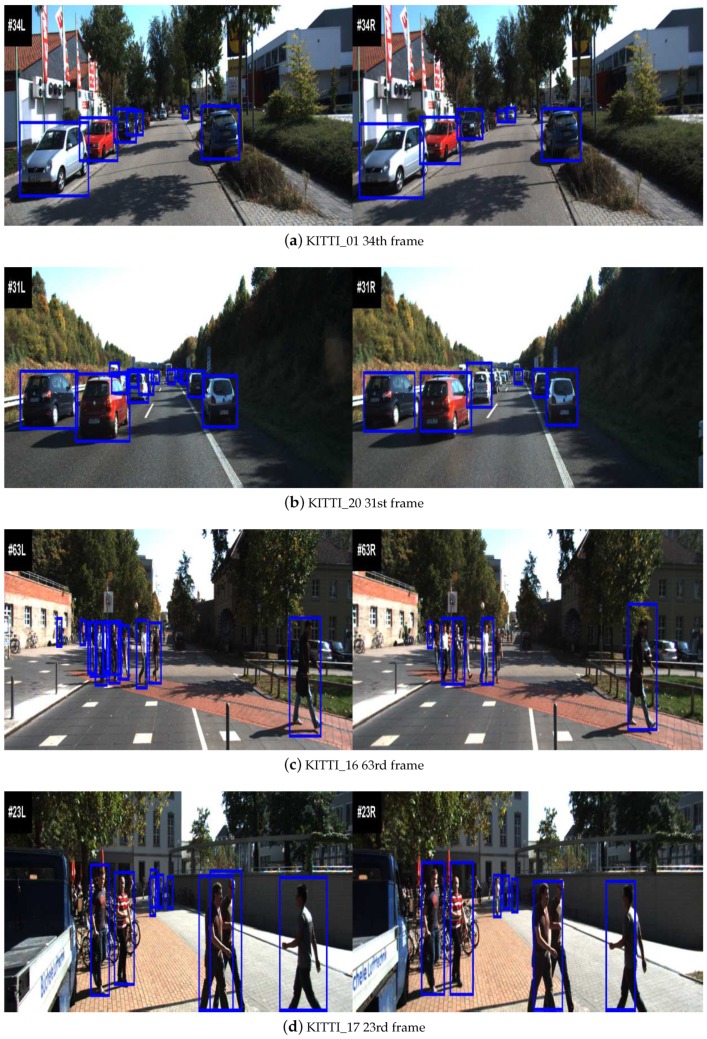
Typical frames of the KITTI dataset [43]. (**a**) Sequence 01 starting frame #34. (**b**) Sequence 20 starting frame #31. (**c**) Sequence 16 starting frame #63. (**d**) Sequence 17 starting frame #23.

In this section, we evaluate the proposed algorithm using the KITTI dataset. Here, the KITTI multi-object tracking dataset [43] is applied to evaluate the proposed data association method. The vehicle tracking test sequences 01 and 20, and pedestrian tracking test sequences 16 and 17 are used. Each sequence consists of 30 frames. Figure 9 depicts the starting frames of left and right cameras for each sequence along with the results of object detection. For the left camera, the detection results of vehicle or pedestrian are provided by the ground truth. Meanwhile, the deformable part model detector method [44] is proposed to detect vehicle or pedestrian for images of rthe ight camera.

**Figure 10:**
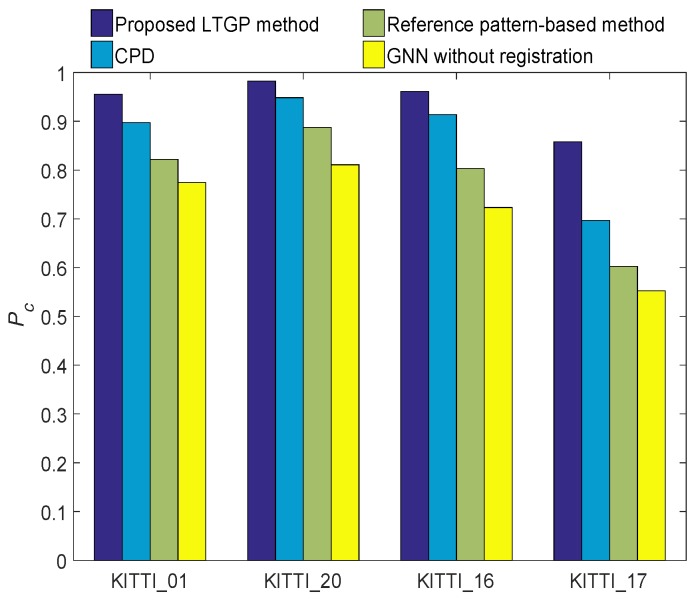
Track-to-track association matching accuracy of the GNN without registration, reference pattern-based algorithm, CPD algorithm and proposed LTGP method under different sequences of KITTI dataset.

The ground truth matching between the left and right images in each frame is confirmed by manual annotation. The GNN without registration, the reference pattern-based algorithm, the CPD algorithm, and the proposed method are employed to associate the local tracks. The average T2TA matching accuracy performances of different T2TA methods are depicted in Figure 10. It is confirmed that the performance of the proposed method is substantially better than those of GNN without registration, the reference pattern-based algorithm, and the CPD algorithm. Compared with the CPD algorithm, the average performance of the proposed method is improved by about 7.8%. In addition, since the KITTI sequence 17 contains large pedestrian occlusion while the motion is more than the other sequences, the performance gap between this and other sequences is more evident. The proposed LTGP method has better performance compared to three benchmark algorithms in the KITTI sequence 17. It is because the proposed method preserves the geometry of local tracks in the data association. The average run-times of these algorithms are given in Table 1, which reveals that the proposed LTGP method has higher computational complexity compared to the GNN without registration, reference pattern-based, and CPD methods.

## 6. Conclusions

A probabilistic method for the track-to-track association, namely, LTGP, was proposed in this paper. In the LTGP method, one local track was transformed into another local track using a nonlinear function. We utilized *k*-connected neighbors to preserve the relative local track geometry. The T2TASB problem was formulated as a probability density estimation problem. The EM algorithm was used to fuse biased tracks from two sensors. To illustrate the advantages of the proposed method, some experiments of computer simulation and KITTI dataset were performed and the result is compared with GNN without registration, reference pattern-based algorithm, and CPD algorithm. Experiments on computer simulation involve varying detection probabilities and different numbers of targets, the proposed method has better performance than other algorithms for all detection probabilities and numbers of targets, but it has higher computational complexity. In the KITTI dataset, the proposed LTGP method has better performance than other methods. The T2TA matching accuracy of the proposed LTGP method was improved by about 7.8% as compared with the CPD method. From the experimental results of computer simulation and KITTI dataset, it can be concluded that the proposed LTGP method outperforms the GNN without registration algorithm, the reference pattern-based algorithm, and the CPD algorithm, but it has a higher computational load.

In the future, the proposed method is not restricted to the considered application but can be extended to other tasks, such as multi-sensor T2TASB for the connected vehicle. For the multi-sensor T2TASB scenario, the LTGP method can be extended using sequential processing.

## Figures and Tables

**Table 1:** The average run-times of these algorithms in four KITTI sequences

	Method	GNN without Registration (s)	Reference Pattern-Based (s)	CPD(s)	LTGP(s)
Sequence					
KITTI_01		0.0004	0.0008	0.0094	0.0159
KITTI_20		0.0004	0.0013	0.0102	0.0169
KITTI_16		0.0008	0.0015	0.0111	0.0176
KITTI_17		0.0004	0.0009	0.0104	0.0163

## Appendix A. The Relationship of Two Local Tracks

Assume that a target is detected by two sensors and that the two sensors are located at the coordinate origins. Due to the sensor bias, the target (x,y) is reported as $x_{t,k}^{1}=\left[x_{t,k}^{1},y_{t,k}^{1}\right]$ and $x_{j,k}^{2}=\left[x_{j,k}^{2},y_{j,k}^{2}\right]$ by the two sensors, respectively. By ignoring the random noises, we have [35]

(A1) $$x_{t,k}^{1}=\left(\Upsilon_{t,k}^{1}+\Delta \Upsilon^{1}\right)\cos\left(\theta_{t,k}^{1}+\Delta \theta^{1}\right),$$

(A2) $$y_{t,k}^{1}=\left(\Upsilon_{t,k}^{1}+\Delta \Upsilon^{1}\right)\sin\left(\theta_{t,k}^{1}+\Delta \theta^{1}\right),$$

(A3) $$x_{j,k}^{2}=\left(\Upsilon_{j,k}^{2}+\Delta \Upsilon^{2}\right)\cos\left(\theta_{j,k}^{2}+\Delta \theta^{2}\right),$$

(A4) $$y_{j,k}^{2}=\left(\Upsilon_{j,k}^{2}+\Delta \Upsilon^{2}\right)\sin\left(\theta_{j,k}^{2}+\Delta \theta^{2}\right),$$

where $\Upsilon_{i,k}^{s}$ and $\theta_{i,k}^{s}$ denote the real range and angle measurement of target *i* for sensor *s*, respectively. The $\Delta \Upsilon^{s}$ and $\Delta \theta^{s}$ represent the range bias and angle bias, respectively. Therefore,

(A5) $$x_{t,k}^{1}\times \frac{\Upsilon_{t,k}^{1}}{\Upsilon_{t,k}^{1}+\Delta \Upsilon^{1}}=x\cos\Delta \theta^{1}-y\sin\Delta \theta^{1},$$

(A6) $$y_{t,k}^{1}\times \frac{\Upsilon_{t,k}^{1}}{\Upsilon_{t,k}^{1}+\Delta \Upsilon^{1}}=x\sin\Delta \theta^{1}+y\cos\Delta \theta^{1},$$

(A7) $$x_{j,k}^{2}\times \frac{\Upsilon_{j,k}^{2}}{\Upsilon_{j,k}^{2}+\Delta \Upsilon^{2}}=x\cos\Delta \theta^{2}-y\sin\Delta \theta^{2},$$

(A8) $$y_{j,k}^{2}\times \frac{\Upsilon_{j,k}^{2}}{\Upsilon_{j,k}^{2}+\Delta \Upsilon^{2}}=x\sin\Delta \theta^{2}+y\cos\Delta \theta^{2}.$$

From (A5)–(A8), we get

(A9) $$\begin{array}{c} \left[\begin{array}{c} x_{j,k}^{2}\\ y_{j,k}^{2} \end{array}\right]=\frac{\Upsilon_{t,k}^{1}\left(\Upsilon_{j,k}^{2}+\Delta \Upsilon^{2}\right)}{\Upsilon_{j,k}^{2}\left(\Upsilon_{t,k}^{1}+\Delta \Upsilon^{1}\right)}\left[\begin{array}{cc} \cos\theta_{12} & \sin\theta_{12}\\ -\sin\theta_{12} & \cos\theta_{12} \end{array}\right]\left[\begin{array}{c} x_{t,k}^{1}\\ y_{t,k}^{1} \end{array}\right] \end{array},$$

where $\theta_{12}=\Delta \theta^{1}-\Delta \theta^{2}$. Equation (A9) gives the relationship between the local tracks $x_{t,k}^{1}$ and $x_{j,k}^{2}$. In this paper, a non-rigid transformation as (5) is proposed to approximate the relationship from one local tracks to other local tracks.

## Abbreviations

T2TA	Track-to-track association
T2TASB	Track-to-track association with sensor bias
LTGP	Local track geometry preservation
GMM	Gaussian mixture model
EM	Expectation-maximization
NN	Nearest neighbor
GNN	Global nearest neighbor
ML	Maximum likelihood
OSPA	Optimal sub-pattern assignment
CPD	Coherent point drift
$X_{k}^{s}$	Local tracks from sensor *s* at time *k*
*K*	Total number of discrete time steps
$N_{k}^{s}$	Number of tracks at time *k* by sensor *s*
$x_{t,k}^{1}$	*t*-th data from sensor 1 at time *k*
$x_{l,k}^{2}$	Centroid of the *l*-th component from sensor 2 at time *k*
N	Gaussian distribution
$\sigma_{k}^{2}$	Equal isotropic covariance at time *k*
*f*	Nonrigid transformation
I	Identity matrix
*D*	Size of a local track vector
$\pi_{t,l}^{k}$	Membership probability of *t*-th row and *l*-th column element in $\pi^{k}$ at time *k*
$\pi^{k}$	Membership probability matrix at time *k*
$Z^{k}$	Indicator matrix
$z_{t}^{k}$	a $1\times N_{k}^{1}$ binary vector for $l=1,2,\ldots N_{k}^{2}$ at time *k*
$z_{t,l}^{k}$	*t*-th row and *l*-th column element in $z_{t}^{k}$ at time *k*
$W_{k}$	an $N_{k}^{2}\times D$ dimensional weight matrix of the Gaussian kernel
$G_{k}$	an $N_{k}^{2}\times N_{k}^{2}$ Gaussian kernel matrix
$g_{ij}$	an *i*-th row and *j*-th column element in $G_{k}$
β	the width parameter in the smoothing Gaussian filter
Tr(.)	Trace of a matrix
L	$N_{k}^{2}\times N_{k}^{2}$ weighted matrix
$L_{lj}$	a *l*-th row and *j*-th column element in L
$G_{k}\left(i,.\right)$	*i*-th row of $G_{k}$
γ	Trade-off parameter controlling between *Q* and E(L)
$R_{k}$	an $N_{k}^{1}\times N_{k}^{2}$ matrix
$C^{k}$	Cost matrix of T2TASB at time *k* as an $N_{k}^{1}\times \left(N_{k}^{2}+1\right)$ matrix
$\left[x_{t,k}^{1},y_{t,k}^{1}\right]$	*x*-axis and *y*-axis positions of target $x_{t,k}^{1}$
$\left[x_{j,k}^{1},y_{j,k}^{2}\right]$	*x*-axis and *y*-axis positions of target $x_{j,k}^{2}$
